# The type IV pili component PilO is a virulence determinant of *Francisella novicida*

**DOI:** 10.1371/journal.pone.0261938

**Published:** 2022-01-25

**Authors:** Mateja Ozanic, Valentina Marecic, Masa Knezevic, Ina Kelava, Pavla Stojková, Lena Lindgren, Jeanette E. Bröms, Anders Sjöstedt, Yousef Abu Kwaik, Marina Santic

**Affiliations:** 1 Faculty of Medicine, Department of Microbiology and Parasitology, University of Rijeka, Rijeka, Croatia; 2 Department of Clinical Microbiology and Laboratory for Molecular Infection Medicine Sweden (MIMS), Umeå University, Umeå, Sweden; 3 Department of Microbiology and Immunology and Center for Predictive Medicine, College of Medicine, University of Louisville, Louisville, Kentucky, United States of America; New York Medical College, UNITED STATES

## Abstract

*Francisella tularensis* is a highly pathogenic intracellular bacterium that causes the disease tularemia. While its ability to replicate within cells has been studied in much detail, the bacterium also encodes a less characterised type 4 pili (T4P) system. T4Ps are dynamic adhesive organelles identified as major virulence determinants in many human pathogens. In *F*. *tularensis*, the T4P is required for adherence to the host cell, as well as for protein secretion. Several components, including pilins, a pili peptidase, a secretin pore and two ATPases, are required to assemble a functional T4P, and these are encoded within distinct clusters on the *Francisella* chromosome. While some of these components have been functionally characterised, the role of PilO, if any, still is unknown. Here, we examined the role of PilO in the pathogenesis of *F*. *novicida*. Our results show that the PilO is essential for pilus assembly on the bacterial surface. In addition, PilO is important for adherence of *F*. *novicida* to human monocyte-derived macrophages, secretion of effector proteins and intracellular replication. Importantly, the *pilO* mutant is attenuated for virulence in BALB/c mice regardless of the route of infection. Following intratracheal and intradermal infection, the mutant caused no histopathology changes, and demonstrated impaired phagosomal escape and replication within lung liver as well as spleen. Thus, PilO is an essential virulence determinant of *F*. *novicida*.

## Introduction

*Francisella tularensis* is a highly virulent intracellular pathogen and the etiological agent of tularemia in animals and humans. The ability to enter, survive and multiply intracellularly is a major virulence mechanism of *F*. *tularensis*, and the strains that have lost this ability are attenuated for virulence [1,2]. *Francisella* uptake and replication occurs in a variety of cell types including phagocytic and non-phagocytic cells. However, it is still not fully known what type of host cells provide *in vivo* growth niches essential for virulence of this bacterium [3–7]. Macrophages are important cells in the pathogenesis of *Francisella* since they are used by the bacterium for intracellular replication and dissemination [8–11].

Interestingly, *F*. *tularensis* also possesses the unique ability to subvert the endosomal lysosomal trafficking pathway within macrophages and modulate host innate response to facilitate its intracellular survival. After engulfment by phagocytic cells, *F*. *tularensis* shortly resides within *Francisella*-containing phagosome (FCP) followed by escape and cytosolic replication [12–14]. The nascent FCP transiently acquires the early endosomal markers (EEA1), which is followed by the expression of late endosomal markers (LAMP-1 and LAMP-2) and the acidification of the phagosome by vacuolar ATPase [15–22]. However, the phagosome does not colocalize with the lysosomal hydrolase cathepsin D [20]. In a period of 30–60 minutes, the phagosomal membrane gets disrupted and bacteria escape to the cytosol of the host cell where bacterial proliferation occurs [12,13,20,23,24]. A brief acidification of the FCP showed to be essential prerequisite for the bacterial escape from the phagosome, since the inhibition of the v-ATPase pump leads to a significant delay of this process [12,20]. Phagosomal escape of the *F*. *tularensis* occurs within multiple cell types including macrophages and neutrophils [14,17,25,26].

To date, very little is known about specific virulence determinants of *F*. *tularensis* and the mechanisms by which bacteria evade the host innate immunity. Factors contributing to virulence include LPS and a capsule [27–30]. In addition, many studies have been performed on the *Francilla* Pathogenicity Island genomic region (FPI), which has been shown to be important for the escape of the bacterium from the phagosome and for subsequent proliferation within the cytosol of host cells.

Studies have shown that the FPI genes encode a non canonical Type VI secretion system (T6SS), which based on a specific gene composition and phylogeny is proposed to represent a unique T6SS subtype [31]. *Francisella* uses the T6SS to translocate effector proteins across bacterial membranes, making this secretion system essential for phagosomal escape and cytosolic replication [32,33].

The transcription of FPI genes is activated by the virulence regulator MglA [4], which is also required for expression of type IV pili (T4P) [34]. T4P are adhesive filamentous surface-located fibers that extend through an outer membrane secretin pore in different bacteria, including *Pseudomonas aeruginosa*, *Neisseria spp*. and *Vibrio cholerae* [35–37]. Genomic analyses have revealed that the genomes of *F*. *tularensis* subsp. *tularensis* strain Schu S4, *F*. *novicida* and *F*. *tularensis* subsp. *holarctica* strain LVS all contain homologues of T4P biogenesis genes [38,39]. In addition, fibers resembling T4P have been visualized on their surfaces [34,38]. Bacteria uses T4P for surface motility, biofilm formation, host cell adhesion, natural transformation or protein secretion [40–42]. The *Francisella* T4P have been shown to contribute to host cell adherence, and are therefore assumed to mediate colonization of different tissues within the host [43]. Similarily, the T4P of *F*. *novicida* have been shown to be involved in secretion of different proteins, including a protease PepO [34,43,44].

Various proteins encoded within specific *pili* clusters on the *Francisella* chromosome are involved in pilus assembly and pilus retraction [34]. One cluster consists of the *pilNOPQ* genes, while another includes *pilFG*. The genes *pilT*, *pilD*, *pilA and pilE* are located elsewhere within the genome [45,46]. Mutations in *pilF* and *pilQ* lead to fewer pili, while *pilA* and *pilF* mutants both exhibit reduced virulence in a mouse model [34,43]. The *pilE1* mutant is attenuated for intramacrophage replication and for virulence in mouse model [34]. In contrast, a strain lacking *pilE4* replicates efficiently within macrophages but is slightly attenuated for virulence in mice [34]. Some of the *pil* genes (*pilB*, *pilC*, *pilQ* and *pilA*) of *F*. *novicida* have been shown to be required for protein secretion in rich medium [47]. The role(s) of *pilO* in the abovementioned processes has yet to be determined, although in *P*. *aeruginosa*, the homologous protein has been shown to be an oligosaccharyl transferase responsible for catalyzing the O-glycosylation of pilin by adding the O-antigen repeating unit to the ß carbon of the C-terminal residue [48].

In this study, a *F*. *novicida pilO* mutant was used to study the role of PilO during *in vitro* and *in vivo* infection models, focusing on the interaction with human monocyte derived macrophages (HMDM) and BALB/c mice. We demonstrate a requirement for PilO in essential processes such as adhesion to the host cells, secretion of effector proteins, as well as phagosomal escape and dissemination, suggesting that PilO is an essential virulence determintant of *F*. *novicida*.

## Materials and methods

### Bacterial strains and growth conditions

Wild type *Francisella novicida* U112 (wt) and a derivative containing a transposon insertion within *pilO* were used in this study [49]. For complementation, *pilO* was expressed from the expression plasmid pKK289Km and introduced *in trans* into the *pilO* mutant, as described previously [50]. Bacteria were grown on buffered charcoal yeast extract (BCYE) agar with 5% CO_2_ for 48h at 35°C, and at 30°C for the visualization of pili.

The expression of the *pilO* in the complemented strain, as well as the expression of neighboring genes (*pilM*, *pilN*, *pilP*, and *pilQ*) in the *pilO* mutant, complemented strain and the wt strain was confirmed. Briefly, bacterial RNA was isolated using RNeasy Protect Mini Kit (Qiagen, Germany), and cDNA was further synthesized using QuantiTect Reverse Transcription Kit (Qiagen, Germany). Gene expression analysis using cDNA was performed with QuantiTect SYBR Green PCR Kit (Qiagen, Germany) and was carried out on the 7500 Fast Real-Time PCR System (Applied Biosystems, USA).

In order to determine the *in vitro* broth culture growth kinetics of the *pilO* mutant, the wt strain and the complemented *pilO/O* strain, we used the Brain Heart Infusion medium (BHI) (Sigma Aldrich, USA). Bacterial suspensions were prepared in BHI at a concentration of 10^2^ CFU/ml and incubated at 35 ºC with 5% CO_2_ for 72 h, with shaking. Bacterial numbers were determined each day by plating the serial dilutions on a buffered charcoal yeast extract (BCYE) agar.

### Isolation and infection of human monocyte-derived macrophages (HMDMs)

Human monocytes were obtained from the whole blood of healthy human volunteers according to the bioethical principles and the law of the Republic of Croatia. Mononuclear cells were isolated by density gradient centrifugation at 4 ºC using Ficoll Paque Plus (GE Healthcare Bio Science AB, Sweden), by a method of adherence within ultra low attachment 6 well-plates (Corning Life Sciences, USA) as previously described (22). During the macrophage isolation, monocytes were grown in RPMI 1640 medium (Thermo Fisher Scientific, USA) with the addition of Fetal Bovine Serum (FBS, Sigma Aldrich) in a decreasing concentrations (from 20% to 1%) during 8 days, to promote the differentiation of monocytes to macrophages. Monocytes were further separated from other mononuclear cells upon growth at 35 ºC with 5% CO_2_.

For growth kinetics assay, cells were incubated in RPMI 1640 medium and infected with *F*. *novicida*, *pilO* mutant and the complemented strain *pilO/O* at multiplicity of infection (MOI) of 100. Cell monolayers were further centrifuged for 5 min at 600 x g at RT and incubated for 1 h at 35 ºC with 5% CO_2_. Cell monolayers were then washed to remove unbound bacteria and treated with 20 μg/ml of gentamicin for 1 h to kill extracellular bacteria, and this was considered as time zero. At each time point after infection (2 h, 24 h, 48 h and 72 h), the cells were lysed using 0.1% Triton-X-100 and the bacterial number was determined by plating 10-fold serial dilutions on BCYE agar.

### Cell adherence assay

For cell adherence assay the HMDMs were infected with bacterial strains (*F*. *novicida* wt, the *pilO* mutant and the complemented strain *pilO/O*) at MOI of 100 and centrifuged for 5 min at 600 x g at RT to facilitate interaction with cells. After 20 min of incubation at 35 ºC with 5% CO_2_, the cells were washed three times using PBS to eliminate unbound bacteria. Further, one set of samples was treated with 0.1% Triton-X-100 to permeabilize the cell membranes and plated to BCYE agar to determine the total number of cell associated bacteria. At the same time, the other set of samples was treated with 20 μg/ml of gentamicin for 1 h, to kill extracellular bacteria. After gentamicin treatment, the cells were lysed using 0.1% Triton-X-100 and plated to BCYE agar to determine the number of intracellular bacteria. The accurate number of adherent bacteria was calculated by deduction of the intracellular bacteria from the total bacterial count.

### Secretion of T6SS effector proteins

The *in vitro* KCl secretion assay was performed as previously described [32]. Briefly, bacteria (*F*. *novicida* wt, the *pilO* mutant, the complemented strain *pilO/O* and the *iglC* mutant as the negative control) were inoculated in Tryptone Soy Broth (TSB) with cysteine and with or without 5% KCl at an initial OD_600_ = 0.03 and grown overnight. After reaching an OD_600_ = 1.5 the samples were pelleted by centrifugation at 4700 x g for 20 min at 4°C. The supernatants were filter-sterilized using 0.45 μm filters and proteins were precipitated using Trichloroacetic acid (TCA). Protein extracts from culture supernatants (1 ml bacterial culture at OD_600_ = 1.5) and bacterial pellets (5x10^7^ bacteria) were prepared using Laemmli sample buffer and immunoblotted. Membranes were probed with mouse anti-PdpB and mouse anti-IglC monoclonal antibodies (BEI Resources, USA) and secondary horseradish peroxidase (HRP)-conjugated anti-mouse antibody (Santa Cruz Biotechnology, USA). The Enhanced Chemiluminescence system (ECL) (Thermo Fisher, USA) was used.

### Mice infections

For testing the virulence of the *pilO* mutant, specific pathogen free, 6–8 weeks old BALB/c mice were used. Mice were treated in accordance to the 3R law regulation procedure Republic of Croatia. Wild type *F*. *novicida*, the *pilO* mutant and the complemented strain *pilO/O* were injected in a volume of 50 μl intradermally (i.d.) or intratracheally (i.t.). Prior to intratracheal infection mice were sedated by an intraperitoneal injection of Xylazine (10 mg/kg) and Ketamine (100mg/kg). I.t. infection was performed by transtracheal instillation, where trachea was exposed surgically on the wentral side of the neck, and a needle was inserted through the tracheal wall into the lumen just bellow the larynx. Bacterial suspension was inoculated in a volume of 50 μl with a 100 μl air pocket behind the inoculum, using 26 Gauge needle and 1 ml syringe.

For survival assay, groups of five mice were infected with different doses of bacteria i.t. or i.d. and monitored daily for a period of 15 days. Actual concentrations of the inoculums were confirmed by plating 10-fold serial dilutions. Within survival study, animals health and behaviour were monitored three times per day, and a humane endpoind was predefined as the end point at which animals were euthanized when they displayed early markers associated with death, including decreased body weight, ruffled fur and behavioural changes. Humane endpoint was applied when animals were found to be in a moribund state with no likehood of recovery. Once animals reached endpoint criteria, no more than few hours elapsed before euthanasia. No animals died before meeting criteria for euthanasia. The animals which survived the 15 days observation period without signs of illness were humanely euthanized at the end of the experiment. Total number of animals used within survival experiment was 115, of which 53 were euthanized at humane endpoint, while 62 were euthanized at the end of the experiment. All animals were euthanized by inducing a deep sedation using a combination of ketamine and xylazine followed by cervical dislocation.

To determine bacterial burden in animal tissues and for histopathology analysis, BALB/c mice were infected with 10 or 10^4^ of bacteria per mouse i.t. or i.d., respectively. At 2 h, 24 h, 48 h, 72 h and 7 days after infection, three mice per group were humanely euthanized. As well as for survival study, within this experiment the humane endpoint was predefined as the point at which animals were euthanized in case they display early markers associated with death. Total number of animals used within this experiment was 90, of which 6 were euthanized at humane endpoint, while 84 were euthanized at certain time point after infection, as planned. At humane endpoint the animals were euthanized by deep sedation using a combination of ketamine and xylazine followed by cervical dislocation. Animals which survived until certain time points were euthanized by intracardial perfusion followed by cervical dislocation. Bacterial loads in the lungs, liver, spleen and kidney were determined. Organs were harvested and homogenized in 5 ml of sterile saline followed by cell lysis in distilled water. Serial 10-fold dilutions were plated on BCYE agar and incubated at 35 ºC with 5% CO_2_. For histopathology assay, lungs, liver and spleen of infected mice were harvested aseptically, fixed in 10% neutral formalin and embedded in paraffin. Tissue samples were further cut in 5 μm sections followed by mounting on a glass slides, staining using haematoxylin and eosin (H&E). The tissue samples were analysed by light microscopy. Research personnel which performed the experiments with mice were specially trained in animal care and use of animals in research.

### Confocal laser scanning microscopy

The adherence of bacterial strains was further followed using confocal microscopy. The HMDMs cells were seeded into 24 well culture plates in a concentration of 10^5^ cells/well, infected with bacterial strains at MOI of 100 and centrifuged at 600 x g, as described above. At 20 min after infection the cells were washed with PBS, fixed using 4% paraformaldehyde (PFA, Sigma-Aldrich, USA) for 30 min at 4°C and permeabilized with 0.1% Triton-X-100. The coverslips were incubated with Anti-*Francisella novicida* monoclonal antibody (Creative Diagnostics, USA), washed with PBS and incubated with Alexa Fluor 555 (Molecular probes, USA) secondary antibodies for 1 h at RT, washed again and mounted using Mowiol (Sigma Aldrich, USA).

In order to study the colocalization of the bacterial strains with early and late phagosomal markers, the HMDMs were again allowed to attach to glass coverslips in 24 well culture plates at a concentration of 10^5^ cells/well. The cells were infected with *F*. *novicida* strains at MOI of 100 for 1 hour at 35°C as previously describd, followed by washing and removal of extracellular bacteria. This was considered as time zero. At different time points after infection (2, 24, 48 and 72 h), the cell monolayers were washed with PBS, fixed for 30 minutes at 4°C with 4% paraformaldehyde and permeabilized by 0.1% Triton-X-100. Anti-*Francisella novicida* antibody (Creative Diagnostics, USA), and EEA1 (Santa Cruz Biotech, USA) or LAMP-1 (Santa Cruz Biotech, USA) antibodies were applied for 1 h at RT. The coverslips were washed with PBS and incubated with Alexa Fluor 555 and Alexa Fluor 488 (Molecular probes, USA) secondary antibodies for 1 hour. The coverslips were further mounted using Mowiol. The analysis was performed using an Olympus FV 1000 confocal microscope.

### Transmission electron microscopy (TEM)

To visualize pili on the surface of *Francisella*, we used a previously described method [51,52]. Briefly, wildtype *F*. *novicida*, the *pilO* mutant, as well as the complemented strain were grown on BCYE agar for 48 h at 30°C. Then, 100 μl of PBS was placed on isolated colonies, and Formvar-coated copper grids (SPI Supplies, USA) were placed gently onto the wetted colonies for 2 min. After that, the grids were removed, and the excess of liquid was wiped off with Whatman grade no. 3 filter paper. Bacteria on the grids were stained with 1% phosphotungstic acid (PTA, Sigma-Aldrich, USA) for 1 min, after which the excess of the PTA was carefully removed with filter paper, and the grids were allowed to air dry for a few minutes. Bacteria were visualized using a transmission electron microscope. Expression of pili was observed in three independent experiments with several hundreds of bacteria being examined.

For observation by electron microscopy, the HMDMs were seeded in 12 well culture plates at 1x10^5^ cell/well and allowed to adhere for a few hours. The cells were infected with *F*. *novicida* strains at a MOI of 100. After 1 h of incubation at 35 ºC with 5% CO_2_, the monolayers were washed with Sorenson’s phosphate buffer to remove extracellular bacteria. This was considered as time zero. At different time points after infection (2, 24, 48 and 72 h), the monolayers were fixed with 2.5% glutaraldehyde and post fixed using 1% osmium tetroxide (OsO_4_). The cells were dehydrated by ethanol series, embedded in epoxy resin (SPI Supplies, USA) and polymerized for 24–48 h at 60 ºC. Ultrathin sections were stained with lead citrate and uranyl acetate and examined by a Phillips Morgany transmission electron microscope.

Animal tissue samples were fixed using 2.5% glutaraldehyde and post fixed with 1% OsO_4_. Samples were dehydrated by acetone series followed by embedding in epoxy resin. Ultrathin sections were stained with lead citrate and uranyl acetate and examined by TEM.

### Statistics

The p values were determined using Pearson’s chi square test or paired two-tailed Student’s t tests. All statistical analyses were performed using GraphPad Prizm software version 6.0 or Statistica software (Statsoft) version 12. Values of p<0.05 were accepted as significantly different in comparison to the *F*. *novicida* wt strain and were labelled with an asterisk*.

## Results

### The PilO protein is important for production of pili on the surface of *F*. *novicida*

Currently, the role of the PilO protein of *F*. *novicida* is not known. In addition, to confirm that the *pilO* transposon mutation did not impact expression of neighboring genes, the expression of neighboring genes (*pilM*, *pilN*, *pilP*, and *pilQ*) in the wt strain, *pilO* mutant and complemented strain was tested using 7500 Fast Real-Time PCR System. Expression level of genes was analyzed relative to wild-type expression using the 2^-ΔΔCT^ method. Although the complemented strain seems to express *pilN* slightly differentially, obtained results showed that the expression of either *upstream* or *downstream* genes was not impaired and that there are no polar effects on the expression of neighboring *pil* genes (see supplemental section S1 Raw data).

**Fig 1:**
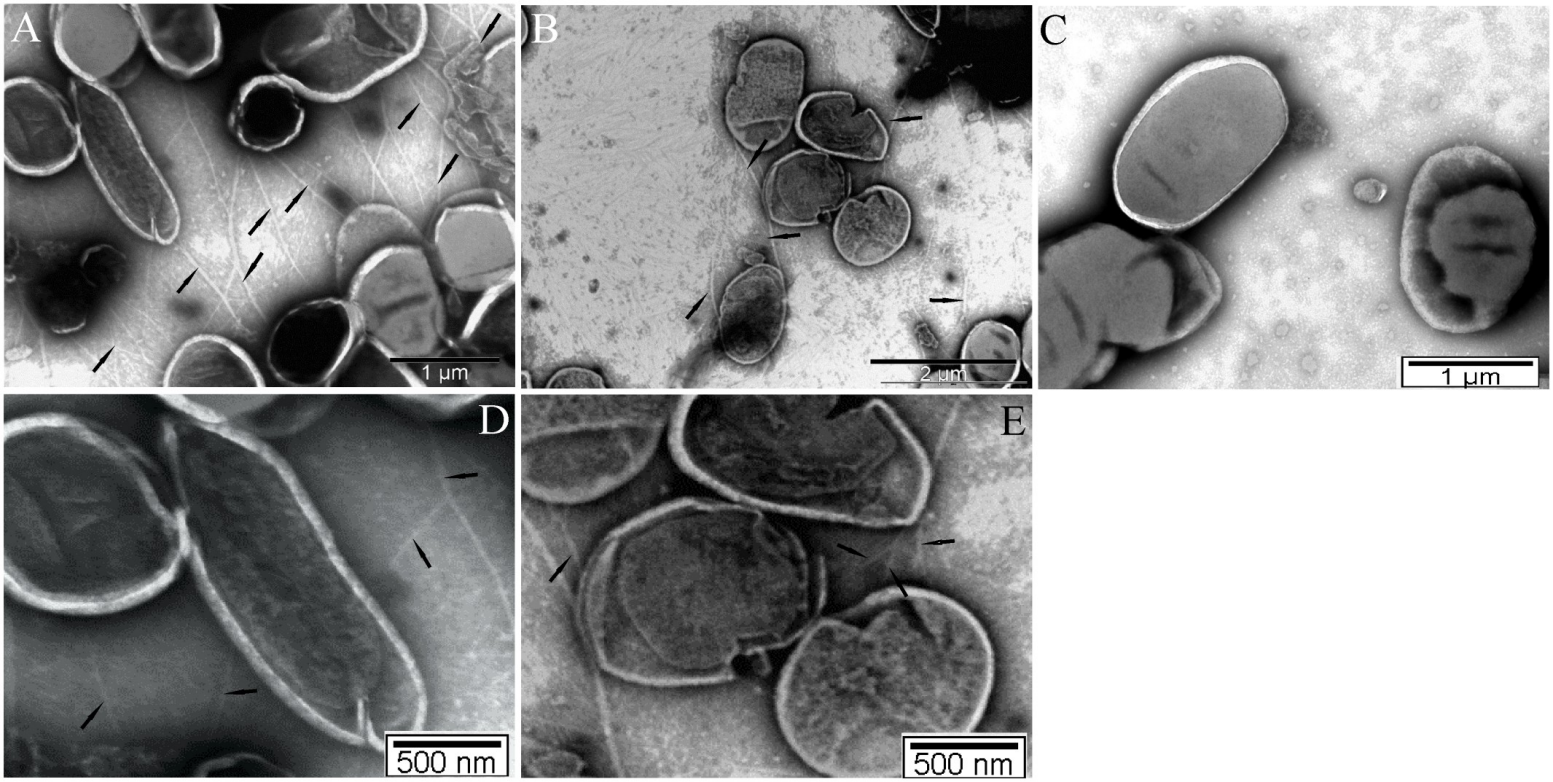
Pili expression on the surface of *F*. *novicida*. Shown are electron micrographs of wt *F*. *novicida* bacteria **(A and D)**, the complemented strain *pilO/O* **(B and E)** and the *pilO* mutant strain **(C)**. On panels A and B, black arrows indicate thick pili structures, while the arrows on panels D and E indicate the thin pili structures. Bacteria were grown at 30°C, stained with PTA and examined by transmission electron microscopy. Pili were observed in three independent experiments, with hundreds of bacteria being examined within each experiment.

To determine whether PilO is required for pilus assembly on the surface of the bacterium, the wt strain of *F*. *novicida*, the complemented strain *pilO/O* and the *pilO* mutant derivative were grown on BCYE agar for 48 h at 30°C and visualized by TEM. On average, ~ 40% of wt bacteria and the complemented strain could be seen to possess pili. Typically, we observed multiple pili radiating from the surface of the wt *F*. *novicida* with a length of 1–2 μm (Fig 1A and 1D). In contrast, the *pilO* mutant bacteria had no detectable pili fibers on the surface (Fig 1C). Similary to the wt strain, around 40% of the complemented strain *pilO/O* showed multiple pili on its surface (Fig 1B and 1E). Both in the wt strain and the complemented strain we could obsere diverse type of pili. On panels A and B the thick type of pili are marked with black arrows, while black arrows on the panels D and E are pointing to the thinner pili of the WT and complemented strain. All bacterial strains showed very similar morphology, as they were present both in a form of cocci and bacilli (Fig 1). Our results indicate that PilO is essential for pili assembly on the surface of *F*. *novicida*.

### The *pilO* mutant shows no defect during *in vitro* growth in broth

**Fig 2:**
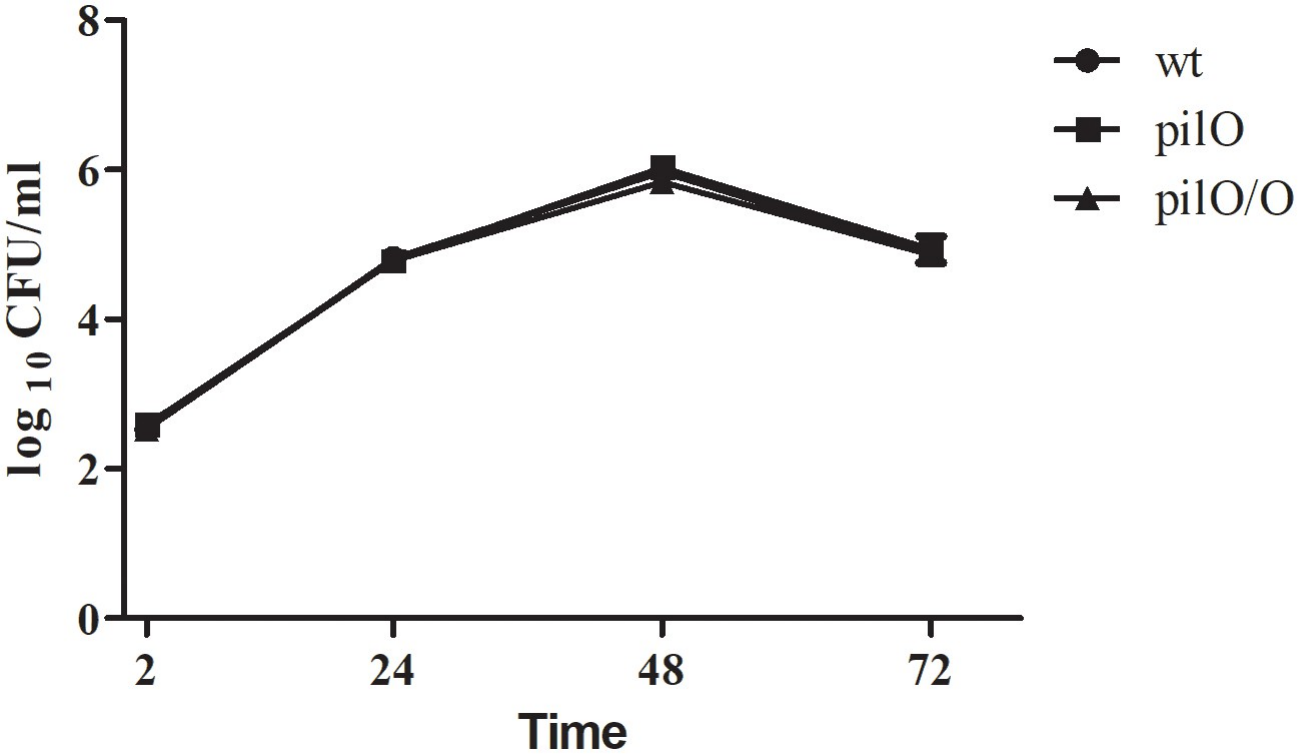
*In vitro* growth kinetics of the *pilO* mutant in BHI medium. Suspensions of 10^2^ CFU/ml of the *pilO* mutant, the wt *F*. *novicida* and the complemented strain were prepared in BHI medium and bacterial numbers were followed during a period of 72 h. Statistical significance was determined by Student t- test (*, p<0.05).

The most commonly used medium for cultivation of *Francisella* is the Brain Heart Infusion medium (BHI). This medium was used to study the growth of the *pilO* mutant in comparison to the wt strain and the complemented strain. Thus, bacterial suspensions were prepared at a concentration of 10^2^ CFU/ml, incubated at 35 ºC and bacterial numbers were followed during a period of 72 h. After an incubation period of 2 h, the numbers of the *pilO* mutant, the wt strain and the complemented strain were close to 5x10^2^ CFU/ml (Fig 2). During an incubation period of 48 h, all bacterial strains reached a concentration around 10^6^ CFU/ml, followed by a slight reduction of bacterial numbers after 72 h (Fig 2). During the whole period, the *pilO* mutant showed no statistical difference in growth kinetics in comparison to the wt strain (Fig 2). Taken together, PilO is redundant for *in vitro* growth of *F*. *novicida*.

### The PilO protein is important for adherence of *F*. *novicida* to host cells

**Fig 3:**
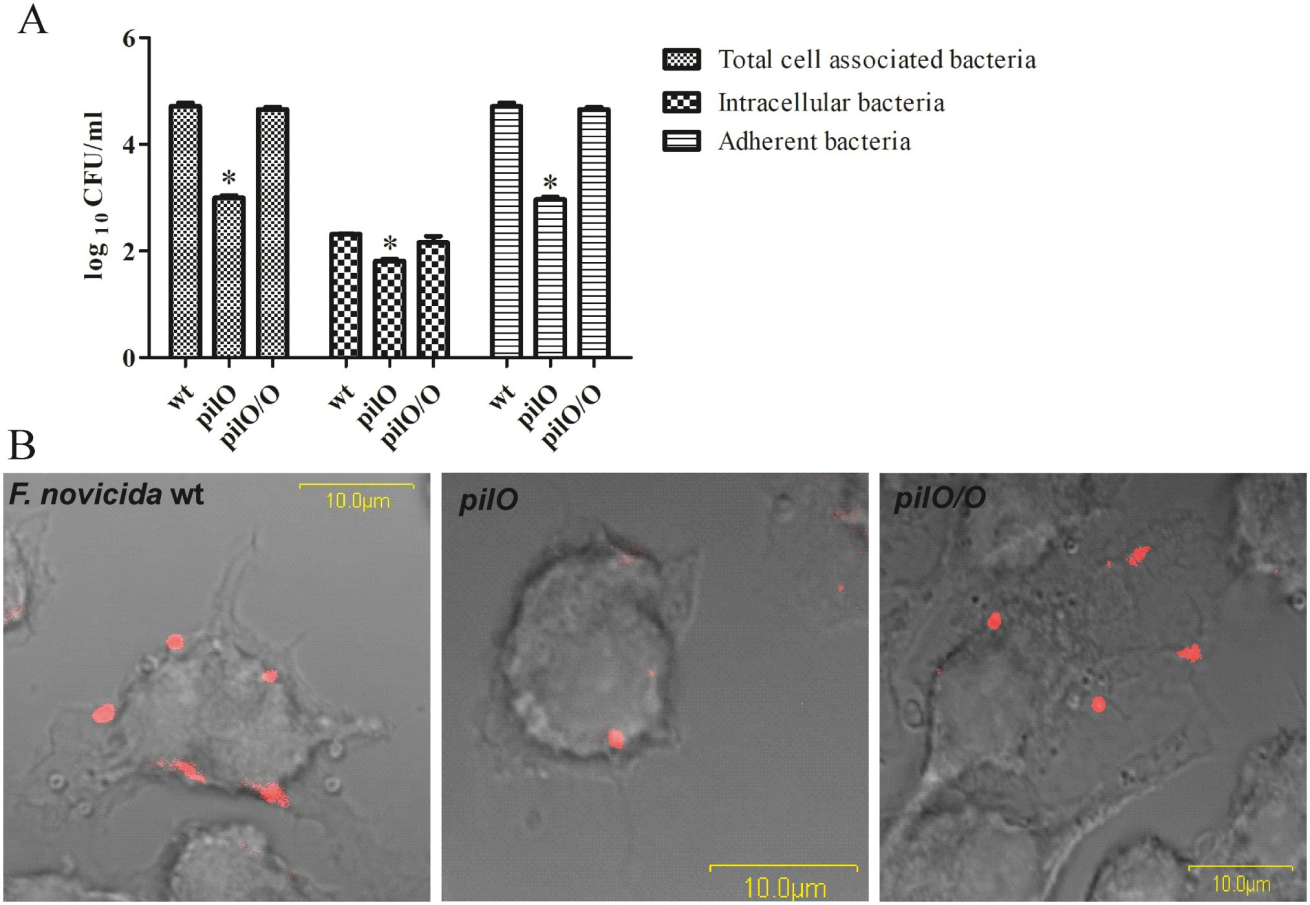
Adherence of the *pilO* mutant to HMDMs. Cells were infected with wt *F*. *novicida*, the *pilO* mutant and the complemented strain at MOI of 100 for 20 min. Unbound bacteria were removed by washing treatment. The number of adherent bacteria **(A)** was calculated by subtraction of the intracellular bacteria from the total number of cell associated bacteria. Confocal microscopy images **(B)** are demonstrating bacteria attached to the surface of HMDMs, where bacteria are labelled red. The images are representative based on analyzing 100 infected cells from three independent experiments. Asterisks denote that the adherence of the *pilO* mutant is statistically different from that of wt *F*. *novicida*, as determined by Student t- test (*, p<0.05).

In order to study the adherence of the *pilO* mutant in comparison to the wt *F*. *novicida*, cell adherence assay was performed with HMDMs cells at 20 min after infection, before the intracellular bacterial replication occures. The total number of cell associated bacteria, as well as the number of intracellular bacteria were determined as described above. The accurate number of adherent bacteria was calculated by deduction of the intracellular bacteria from the total bacterial count (Fig 3A). At the same time point, at 20 minutes after infection, the adherence of bacteria was followed using confocal microscopy (Fig 3B).

Within this experiment, the *pilO* mutant showed significantly lower ability to adhere to the surface of HMDMs, in comparison to the wt strain. At 20 min after infection the total number of the wt *F*. *novicida* associated with the cells was 7x10^4^ CFU/ml while at the same time only 10^3^ CFU/ml of the *pilO* mutant associated with the HMDMs (p< 0.05) (Fig 3A). The number of intracellular bacteria at that time was only around 200 CFU/ml of the wt strain and 65 CFU/ml of the *pilO* mutant (Fig 3A). Hence, at 20 min after infection, the majority of detected bacteria were attached to the surface of the HMDMs. Hundred cells per sample were analysed using the confocal microscopy to further compare the adherence of the *pilO* mutant and the wt strain. In average we detected 5 of the wt bacteria and 1 of the *pilO* mutant attached to the HMDMs cell (p< 0.05) (Fig 3B), which is consistent with the results shown within Fig 3A. At the same time, the complemented *pilO/O* strain showed wery similar behaviour to the wt strain (p>0.05) (Fig 3A and 3B).

### PilO plays a role in intracellular replication of *F*. *novicida* within HMDM cells

*F*. *tularensis* possesses the ability to subvert the endosomal lysosomal trafficking pathway within macrophages. After engulfment by phagocytic cells, *F*. *tularensis* shortly resides within an FCP followed by disruption of the phagosomal membrane, bacterial escape and cytosolic replication [3,12–14,17,26]. We examined intracellular growth kinetics of the *pilO* mutant of *F*. *novicida* in HMDMs in comparison to the wt *F*. *novicida* and the complemented strain *pilO/O*. The cells were infected with the *F*. *novicida* strains at MOI of 100 and the number of intracellular bacteria was determined at 2, 24, 48 and 72 h after infection.

**Fig 4:**
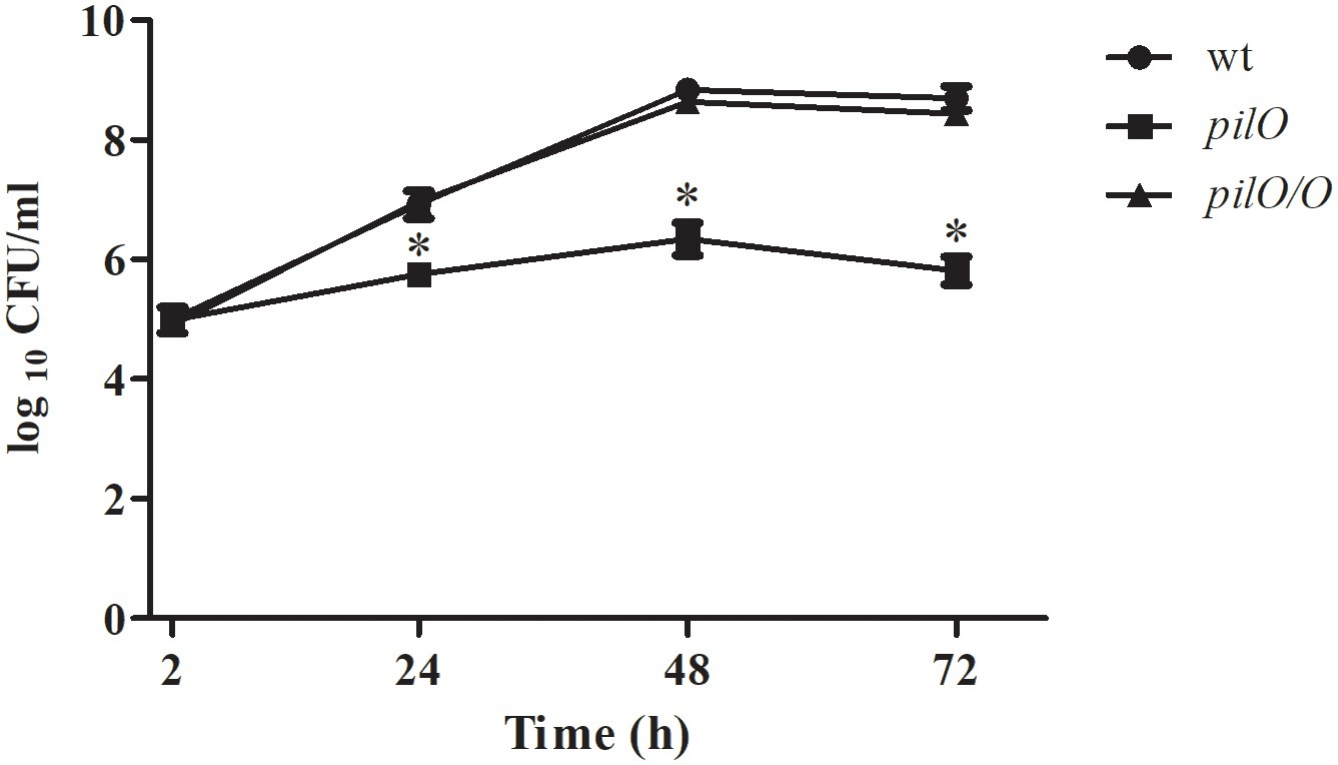
Growth kinetics of the *pilO* mutant within HMDMs. Cells were infected with wt *F*. *novicida*, the *pilO* mutant and the complemented strain at MOI of 100. Growth kinetics was determined at 2, 24, 48 and 72 h after infection. Extracellular bacteria were removed by washing. The error bars represent standard deviations of three independent biological replicates. Asterisks denote that the growth kinectics of the *pilO* mutant is statistically different from that of wt *F*. *novicida*, as determined by Student t- test (*, p<0.05).

As expected, the wt *F*. *novicida* replicated efficiently within HMDM cells. Already at 2 h after infection, the number of intracellular *F*. *novicida* was 10^5^ CFU/ml (Fig 4). At the same time, the number of intracellular *pilO* mutant bacteria within HMDMs was 9x10^4^ CFU/ml, which was not significantly different from the count of the wt strain (p>0.05) (Fig 4). The number of intracellular wt *F*. *novicida* bacteria increased to 9x10^6^ CFU/ml at 24 h and it reached peak of replication at 48 h when the bacterial number was 7x10^8^ CFU/ml (Fig 4). By 72 h the bacterial count of the wt strain in HMDMs slightly declined to 5x10^8^ CFU/ml (Fig 4). In contrast, at the same time points we noticed significant decreases in the bacterial counts of the *pilO* mutant. At 24 h there was 7x10^5^ CFU/ml of the intracellular *pilO* mutant strain (p<0.05), and by 48 h the number of *pilO* was 2x10^6^ CFU/ml (p<0.05) (Fig 4). By 72 h, the bacterial count of *pilO* mutant strain slightly declined to 7x10^5^ CFU/ml (p<0.05) (Fig 4). During the observed period, the complemented strain *pilO/O* showed very similar growth kinetics as the wt strain (p>0.05) (Fig 4). Altogether, compared to the wt strain, the *pilO* mutant showed a major defect in replication within HMDM cells.

### Loss of PilO affects T6SS effector proteins

It is well known that the intracellular life cycle of *Francisella* is supported by the FPI encoded T6SS, which injects the effector proteins into the host cell and enables bacterial escape from the phagosome [32,53,54]. Although there are fundamental differences in the T6SS among the *Francisella* species, the secretion of effector proteins PdpA, IglC, IglE and PdpE was detected by both *F*. *tularensis* and *F*. *novicida* [54,55]. However, the molecular mechanisms of the T6SS function are still largely unknown and the involvement of the PilO protein in the T6SS process has not been described yet.

**Fig 5:**
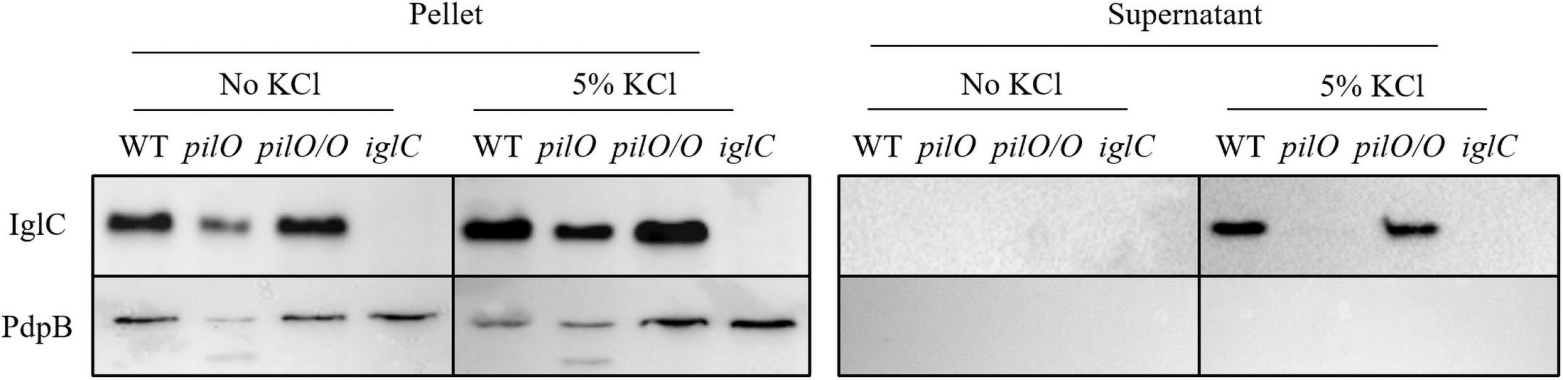
Secretion of the T6SS effector proteins. The presence of the IglC was followed in the concentrated supernatants or in bacterial pellets of wt *F*. *novicida*, the *pilO* mutant and the complemented strain *pilO/O* grown in the presence or absence of 5% KCl. As control, a secretion defective mutant, the *iglC*, was used as well as the inner membrane protein the PdpB. Figure shows one experiment representative of three independent experiments.

To test our hypothesis that deletion of *pilO* may affect the secretion of effector proteins by *F*. *novicida*, we used an *in vitro* secretion assay in which addition of KCl triggers T6SS-dependent secretion [32]. Our results show that the IglC protein is secreted into culture supernatants of *F*. *novicida* and complemented strain *pilO/O* upon KCl treatment, but not in the supernatant of the *pilO* mutant (Fig 5). As expected, the inner membrane protein PdpB was not detected in the culture supernatants (Fig 5). Also, the IglC and PdpB proteins were not detected in culture supernatants when bacteria were grown without KCl (Fig 5). The levels of IglC and PdpB were also reduced in the pellet fraction (Fig 5), indicating that loss of PilO results in decreased levels of these T6SS proteins in the bacteria. (See supplemental section S2 and S3 Raw data).

### Failure of the *pilO* mutant to escape from the FCP within HMDMs

Shortly after engulfment by phagocytic cells, the FCP transiently acquires the early endosomal markers (EEA1), followed by the acquisition of the late endosomal markers (LAMP-1 and LAMP-2) as well as the vacuolar ATPase which acidifies the phagosome [15–18,20–22,56]. This is followed by the disruption of the FCP, and escape of the *Francisella* into the cytosol of the HMDMs where bacterial proliferation occurs [20,57]. To determine whether the *pilO* mutant strain is defective for phagosomal escape, we infected HMDMs with the *pilO* mutant or the *F*. *novicida* wt strain. Confocal microscopy was used to investigate bacterial trafficking along the endocytic pathway within the cells and colocalization of bacteria with EEA1 and LAMP-1 markers.

**Fig 6:**
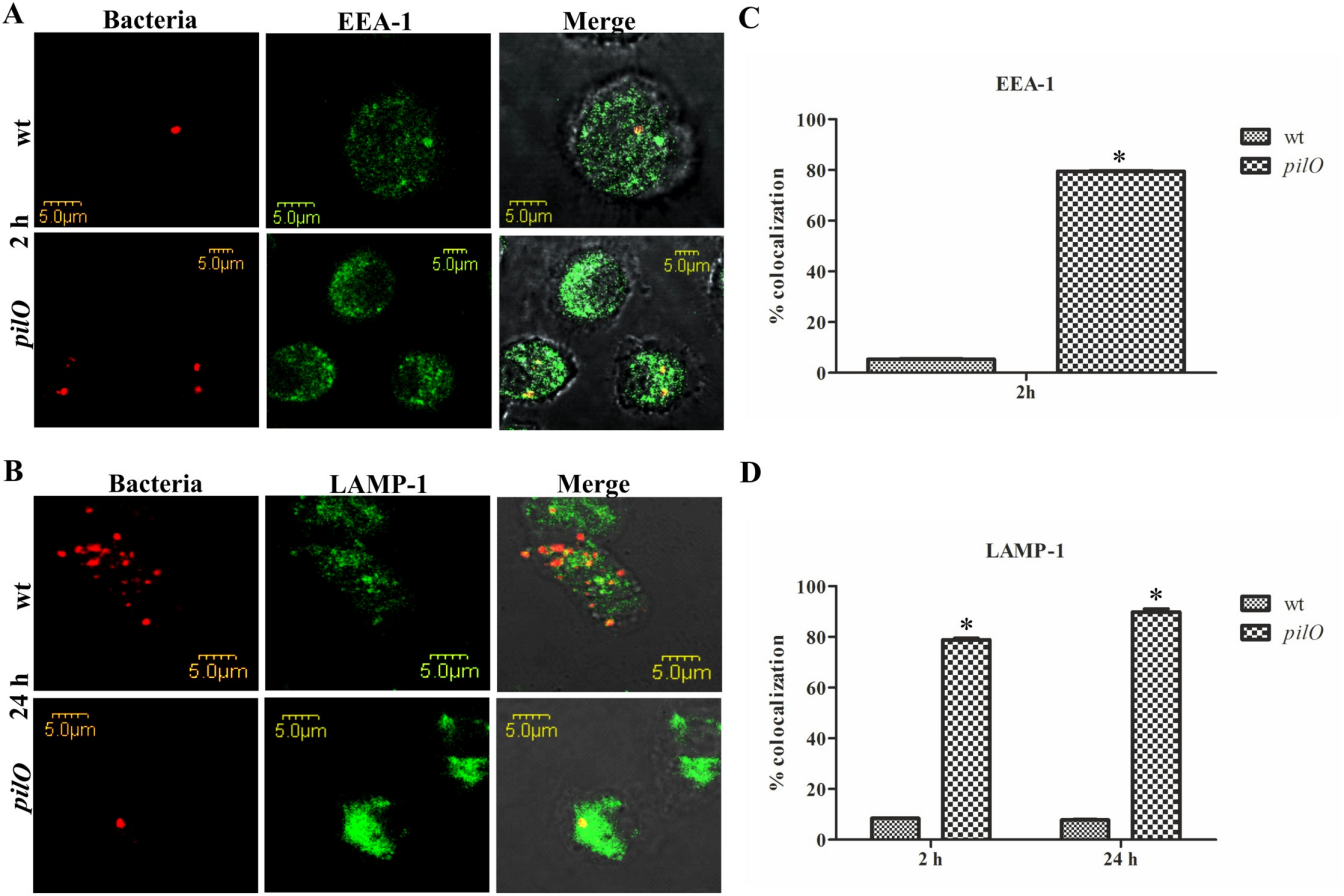
Trafficking of the *pilO* mutant strain within HMDMs. Shown are representative confocal laser scanning microscopy images, demonstrating the colocalization of wt *F*. *novicida* or the *pilO* mutant with the endosomal markers EEA-1 **(A)** or LAMP-1 **(B)**, where bacteria are labelled red and the markers green. Quantitative analyses of the EEA-1 **(C)** and LAMP-1 **(D)** expression by the FCPs within HMDMs. All results are based on analyzing 100 infected cells from three coverslips, with the error bars representing the standard deviations. The results shown within panels A and B are a representative of three independent experiments, while the panels C and D are showing the average data of three independent experiments. Asterisks denote that the colocalization of the *pilO* mutant is statistically different from that of wt *F*. *novicida*, as determined by Student t- test (*, p<0.05).

At 2 h after infection with the wt strain, the colocalization of the FCP with the early endosomal marker EEA1 was only 5.4% (Fig 6A and 6C) and with LAMP-1 8.51% (Fig 6D), indicating that the majority of *F*. *novicida* were either in the cytosol or in a compartment lacking these markers. In contrast, at the same time point, 79.5% of the *pilO* containing phagosomes colocalized with the EEA1 marker (p<0.05) (Fig 6A and 6C) and 78.9% associated with LAMP-1 (p<0.05) (Fig 6D), indicating a lack of bacterial phagosomal escape.

Abundant intracellular replication of the wt strain was observed at 24 h after infection. At the same time, 89.9% of intracellular *pilO* mutants were localized in a LAMP-1 positive compartments (p<0.05) (Fig 6B and 6D). These results demonstrate that within the HMDMs, the *pilO* mutant fails to escape the FCP.

### Attenuation of the *pilO* mutant for virulence in BALB/c mice

Clinical manifestation of tularemia depends on the infection dose as well as the route of infection [58]. While respiratory infections cause the most severe form of the disease, the i.d. route is considered as the natural and the most common route of infection.

In order to determine the LD_50_ for the i.t. or i.d. routes of infection, five BALB/c mice per group were inoculated with the *pilO* mutant strain and the course of infection compared to the wt strain as well as a complemented strain, All animals were monitored daily for survival during 10 days.

**Fig 7:**
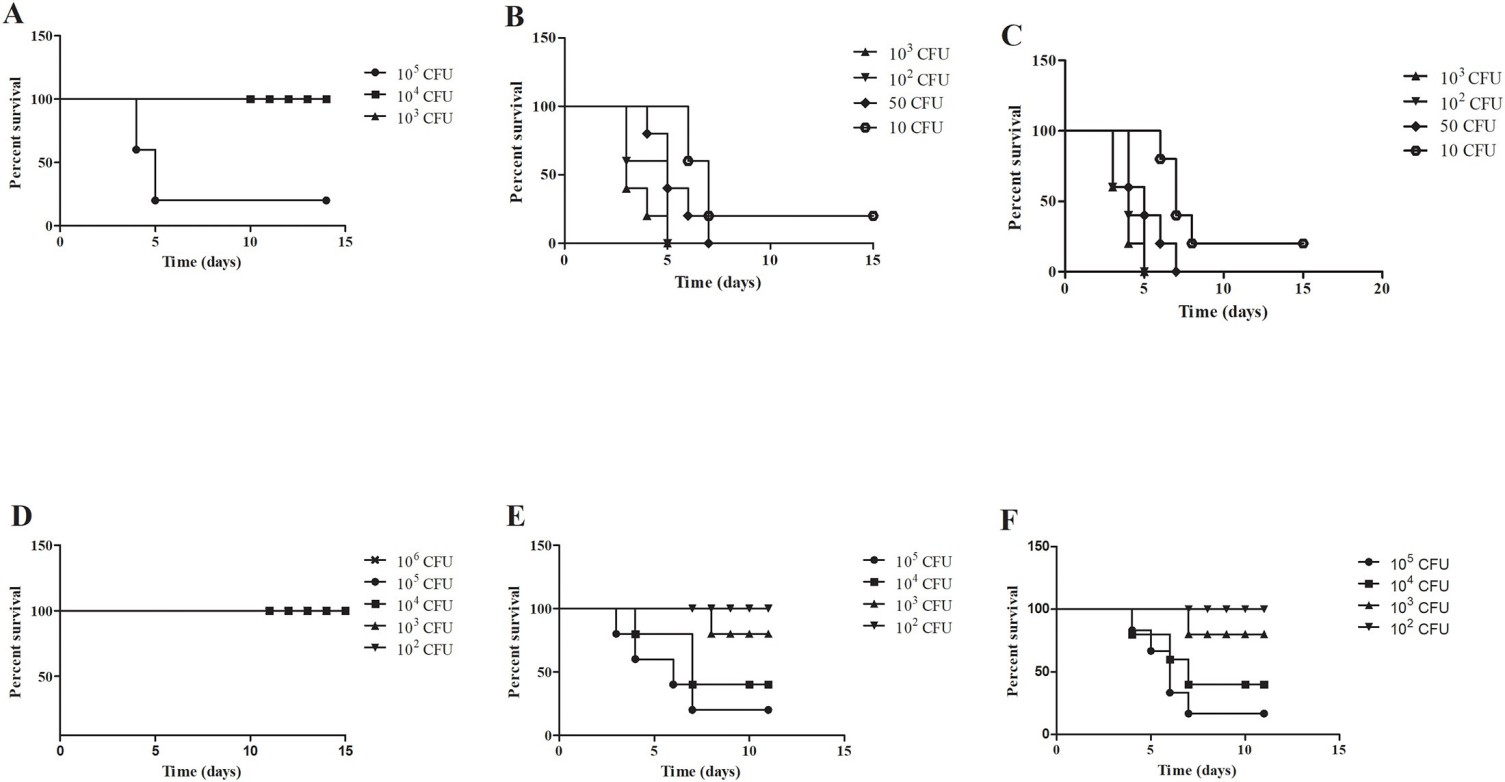
Survival of BALB/c mice infected with *F*. *novicida*. Groups of 5 mice were infected intratracheally (i.t.) with the *pilO* mutant strain **(A)**, wt *F*. *novicida* **(B)**, and the *pilO*/*O* complementant **(C)**. In addition, 5 mice per group were infected intradermally (i.d.) with the *pilO* mutant strain **(D)**, wt *F*. *novicida* **(E)** and the *pilO*/*O* complementant **(F)**. The infection doses are indicated in the figure legends. Infected animals were monitored for 15 days. The Prism GraphPad5 software was used to generate graphs as well as for the statistical analysis.

Intratracheal (i.t.) infection of mice with the *pilO* mutant strain resulted in 100% survival with the doses of 10^2^, 10^3^ and 10^4^ CFU per mouse, whereas the survival rate of animals infected with 10^5^ CFU/mouse was 20% (Fig 7A) (for all applied doses p<0.05). Mice succumbed to infection after 4 or 5 days (Fig 7A). All BALB/c mice infected i.t. with the wt strain using a dose of 10^2^ or 10^3^ CFU/mouse died within 3 to 5 days post infection (Fig 7B). Mice infected with 50 CFU of the wt strain also showed no survival and died within 4–7 days (Fig 7B). Following i.t. infection with 10 CFU of the wt strain, the survival rate was 20% and mice started to die at day 6 (Fig 7B). Regardless of dose, i.t. infection with the complemented strain *pilO/O* resulted in very similar results as for the wt *F*. *novicida* (Fig 7B and 7C) (for all applied doses p>0.05).

Intradermal (i.d.) infection of BALB/c mice with all doses of the *pilO* mutant strain tested (10^2^, 10^3^, 10^4^, 10^5^ and 10^6^ CFU/mouse) resulted in a 100% survival rate (Fig 7D). Intradermal infection of BALB/c mice using the dose of 10^2^ CFU/mouse of wt *F*. *novicida*, resulted in 100% survival rate during ten days observation (Fig 7E), while doses 10^3^ CFU/mouse and 10^4^ CFU/mouse, resulted in survival rates of 80% and 40% respectively (Fig 7E). Animals died between 3 and 10 days after infection (Fig 7E). Following i.d. infection with 10^5^ CFU/mouse of wt *F*. *novicida*, 20% of infected animals survived during ten days of observation (Fig 7E) (p>0.05 for the dose of 10^2^ and p*<*0.05 for all other applied doses). A similar trend was also observed for the *pilO/O* complemented strain (for all applied doses p>0.05). Thus, all animals survived i.d. infection with 10^2^ CFU of *pilO/O*, while animals infected with 10^3^ or 10^4^ CFU/mouse showed survival rates of 80% and 50%. As for the wt strain, animals infected with the *pilO/O* started to die at day 3 after infection (Fig 7F). Following i.d. infection with 10^5^ CFU/mouse of *pilO/O*, 20% of infected animals survived during ten days of observation (Fig 7F). Thus, complementation of the *pilO* mutant *in trans*, efficiently restores the ability of the bacteria to cause disease.

The results show that LD_50_ of the wt strain is 10–50 CFU by the i.t. route, and 10^4^ CFU by the i.d. route. In contrast, the LD_50_ of the *pilO* mutant strain is around 10^5^ CFU following i.t. infection, while no mouse died from the same dose upon i.d. infections. This suggests that mice are more susceptible to i.t. than i.d. infection with *F*. *novicida* strains, and, importantly, that the *pilO* mutant is attenuated for virulence in BALB/c mice, regardless of the route of infection.

### Attenuation of the *pilO* mutant for replication and dissemination within internal organs of BALB/c mice

To further study the virulence of the *pilO* mutant, the BALB/c mice were infected by i.t. and i.d. route and the bacterial burdens within lungs, liver and spleen were analysed.

In order to determine the growth kinetic of the *pilO* mutant in lung, liver, spleen and kidney, three BALB/c mice per group were infected i.t. (10 CFU/mouse) or i.d. (10^4^ CFU/mouse) using *F*. *novicida* wt or the *pilO* mutant strain. Regardless of route, lung, spleen, liver and kidney were harvested from infected mice at 2 h, 24 h, 48 h, 72 h and 7 days after infection and plated to determine individual organ burden.

**Fig 8:**
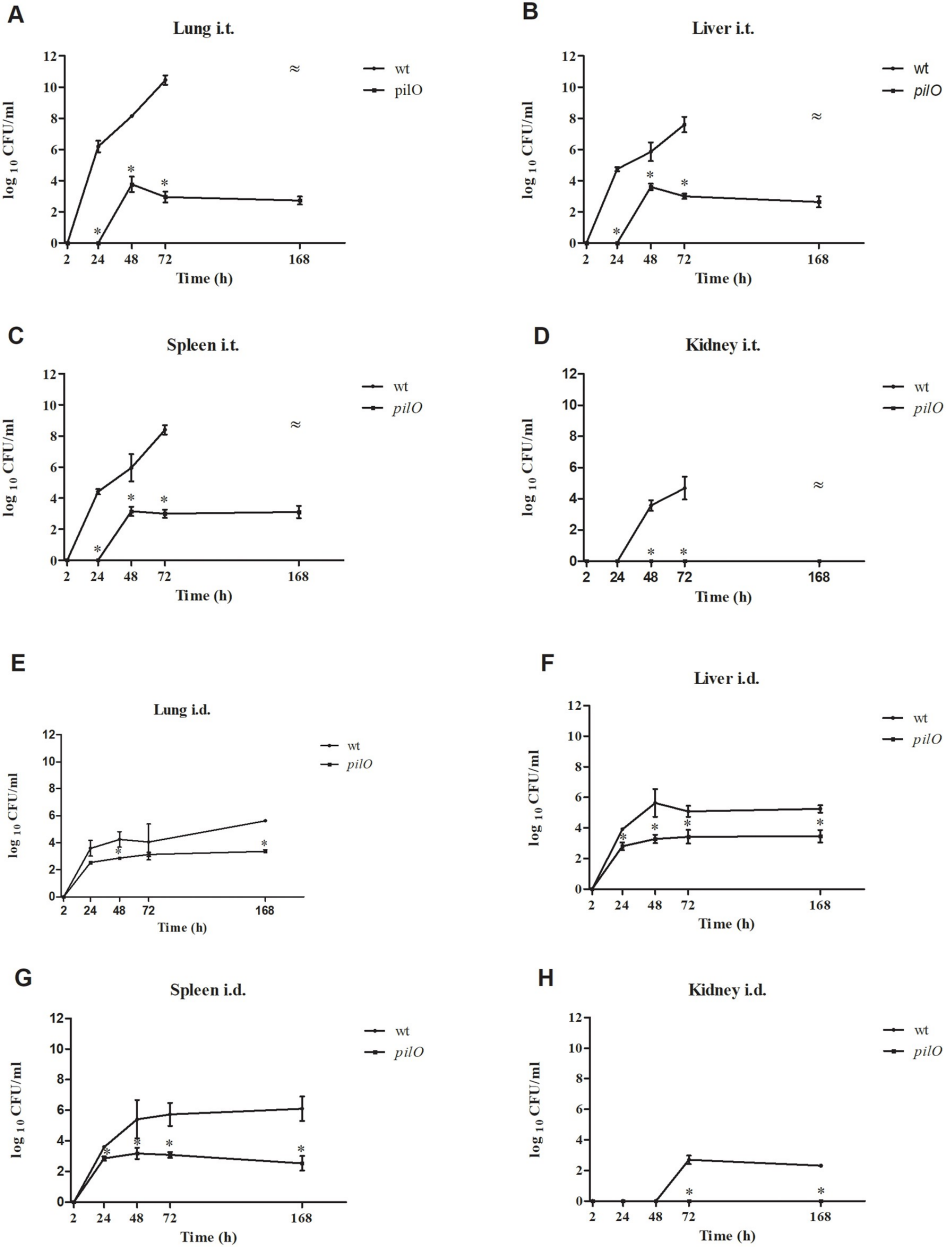
Growth kinetics of *F*. *novicida* within the organs of infected BALB/c mice. Groups of three mice were infected intratracheally (i.t.) with 10 bacteria per mouse using wt *F*. *novicida* or the *pilO* mutant strain. Bacterial burden was determined in lung **(A)**, liver **(B)**, spleen **(C)** and kidney **(D)** at 2 h, 24 h, 48 h, 72 h, and 7 days after infection. Other groups of three mice were infected intradermally (i.d.) with 10^4^ bacteria per mouse, using the identical strains and time points, upon which the bacterial burden was determined in lung **(E)**, liver **(F)**, spleen **(G)** and kidney **(H)**. The error bars represent standard deviations of triplicate samples. **≈** Bacterial burdens for mice infected with the wt *F*. *novicida* were not determined at 7 days after i.t. infection because the mice did not survive until that time point. Asterisks denote that the growth kinetics is statistically different from that of wt *F*. *novicida*, as determined by Student t- test (*, p<0.05).

By the i.t. route, *F*. *novicida* wt was find to replicate efficiently in the lung, liver, spleen and kidneys of BALB/c mice, while the *pilO* mutant strain showed no replication in tested organs (Fig 8A–8D). By 72 h after infection, the number of wt bacteria reached 4x10^10^ CFU/ml in the lungs (Fig 8A), 6x10^7^ CFU/ml in the liver (Fig 8B) and 4x10^8^ CFU/ml in the spleen (Fig 8C). Bacterial count of the wt strain in the kidney was lower in comparison to other organs, but it still reached 7x10^4^ CFU/ml at 72 h (Fig 8A–8D). Since mice infected with wt *F*. *novicida* died before 7 days upon i.t. inoculation, bacterial burden was not determined at this time point. In contrast, the *pilO* mutant was not detected in the lung, liver and spleen at 2 and 24 h, while by 72 hours p.i. the bacterial number was 1x10^3^ CFU/ml in lungs (p<0.05) (Fig 8A), 2x10^3^ CFU/ml in the liver (Fig 8B) (p<0.05), and 3x10^3^ CFU/ml in the spleen (p<0.05) (Fig 8C), while the *pilO* mutant strain was not found in the kidney at any time point after infection (p<0.05) (Fig 8D). At 7 days post infection, the mutant was still detected in all other organs (Fig 8A–8C).

Following i.d. infection, *F*. *novicida* wt was shown to replicate in the lung, liver, spleen and kidney of infected animals, while the replication of the *pilO* mutant was significantly reduced (p<0.05) (Fig 8E–8H). The number of wt in lungs reached a peak of 6x10^5^ CFU/ml at 7 days post infection (Fig 8E). In contrast, the number of the *pilO* mutant strain in lungs was constantly 1–2 logs lower during the observed period reaching the 1x10^3^ at 72 h, (p<0.05) (Fig 8E). Bacterial counts of the wt in liver of mice reached a peak at 48 h (6x10^5^ CFU/ml) and slightly declined by 7 days (Fig 8F) (2x10^5^ CFU/ml). However, during the observed period, the number of the *pilO* mutant strain in the liver was constantly 2 log lower in comparison to the wt strain (p<0.05) (Fig 8F). The number of the wt strain in spleen was increasing during the observed period, reaching 10^6^ CFU/ml at day 7 after infection (Fig 8G). In contrast, the number of the *pilO* mutant in spleen was roughly 1–2 logs lower during the observed period, reaching the maximum values of 2x10^3^ at 48 h after infection (p<0.05) (Fig 8G). *F*. *novicida* wt was not found in the kidney at 2, 24 and 48 h, but at 72 h there were 7x10^2^ CFU/ml of bacteria (Fig 8H). The *pilO* mutant strain was not detected in the kidney at any time point (p<0.05 at 72 h and 7 days p.i.) (Fig 8H).

Altogether, these results clearly demonstrate that the *pilO* mutant is attenuated for dissemination and replication within the organs of BALB/c mice, including lung, liver and spleen. Results are consistent regardless of the route of inoculation, i.e. intratracheal or intradermal infection.

### Pathological changes in organs of BALB/c mice induced by the *pilO* mutant

**Fig 9:**
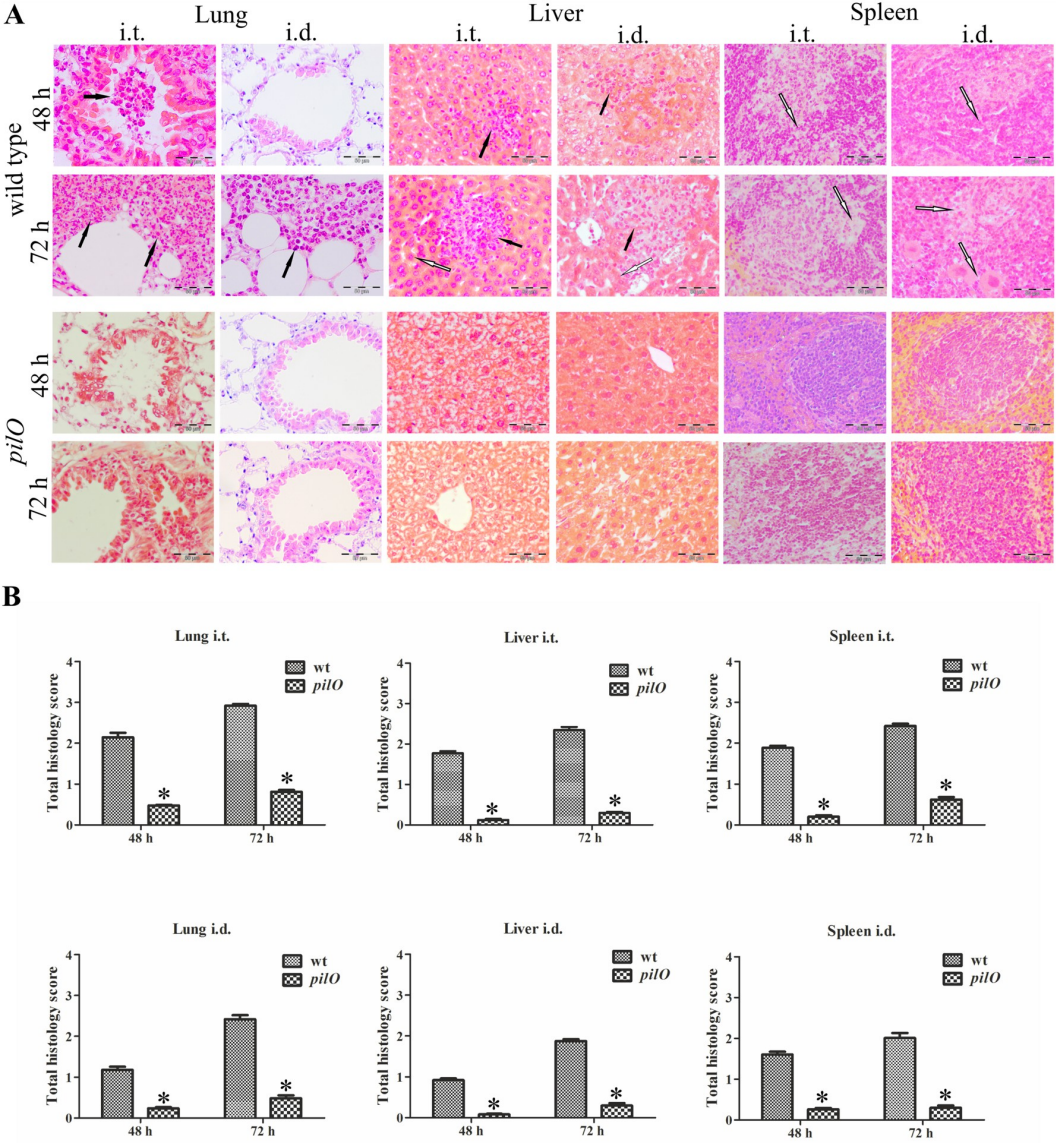
Histopathology of mouse organs infected with *F*. *novicida*. Mice, three per group, were infected via the intratracheal (i.t.) or intradermal (i.d.) route using wt *F*. *novicida* or the *pilO* mutant. At 2 h, 24 h, 48 h and 72 h after infection, tissue sections from lungs, liver and spleen were stained with H&E and analysed by light microscopy **(A)**. Representative images of twenty microscopic fields were taken for each sample. Black arrows point the presence of inflammatory infiltrates, while white arrows indicate the destruction of parenchyma. Histopathology scoring of lungs, liver and spleen tissue **(B)**. Twenty high-powered fields were randomly chosen to grade the severity of inflammation as well as the overall damage of organs parenchyma. The histology assessment consists of grading the presence of inflammatory infiltrates and affected parenchyma. Lung tissue was scored for infiltration of mononuclear cells and alveolar and bronchial involvement, liver tissue was scored for hepatocyte vacuolation and the presence of inflammatory infiltrates, while the spleen tissue was scored for overall destruction of architecture of red pulp, white pulp and capsule. The total histology score of each organ was calculated as an average of individual criteria scores (0-absent, 1-slight, 2-moderate, 3-severe). The error bars show the standard deviations. Asterisks denote that the histopathology scoring of the *pilO* mutant is statistically different from that of wt *F*. *novicida*, as determined by Student t- test (*, p<0.05).

To further study the virulence of the *pilO* mutant, histopathological changes within lung, liver and spleen of infected mice were analysed. Total histopathology score of each organ was calculated as an average of the individual criteria scores. The occurance of inflammatory infiltrates and affected parenchyma were determined for lungs, liver and spleen. At all observed time points after i.t. or i.d. infection with the wt strain, we observed the infiltration of mononuclear cells within peribronchial spaces, bronchiole and alveoli as well as a severe lung parenchyma destruction (Fig 9A and 9B). In the group of mice inoculated with the *pilO* mutant, no histopathological changes were observed until 72 h. At that time point, we noticed some infiltration of mononuclear cells, however with significantly less intensity in comparison to the wt strain (i.t. p*<*0.05 and i.d. p*<*0.05) (Fig 9A and 9B). Within liver tissue, at 48 and 72 h after i.t. and i.d. infection with the wt, there were numerous areas of inflammatory infiltrates as well as hepatocyte degeneration. In contrast, no histopathologic changes were observed in animals infected with the *pilO* mutant regardless of infection route (i.t. p<0.05, i.d. p*<*0.05) (Fig 9A and 9B). Similarly, the wt strain caused severe destruction of the spleen tissue, including the damage of the white, red pulp and the capsule. In contrast, infection of mice with the *pilO* mutant did not cause any damage of the spleen tissue structure (i.t. p<0.05, i.d. p<0.05) (Fig 9A and 9B). Altogether, the *pilO* mutant caused no significant histopathology changes within organs of infected mice, consistent with its defect in dissemination and intracellular replication.

### Failure of the *pilO* mutant to escape from the FCP of infected organ cells during mice infections

Little is known about the importance of *pil* genes for the intracellular localisation of *F*. *novicida* in mice. In order to study the role of the *pilO* gene, the BALB/c mice were infected with wt *F*. *novicida* or the *pilO* mutant strain using 10 CFU/mouse (i.t. infection) or 10^4^ CFU/mouse (i.d. infection). At 72 h after infection, lungs and spleens of infected mice were examined by TEM for the localization of bacteria within the host cells, as well as the integrity of the phagosomal membrane.

**Fig 10:**
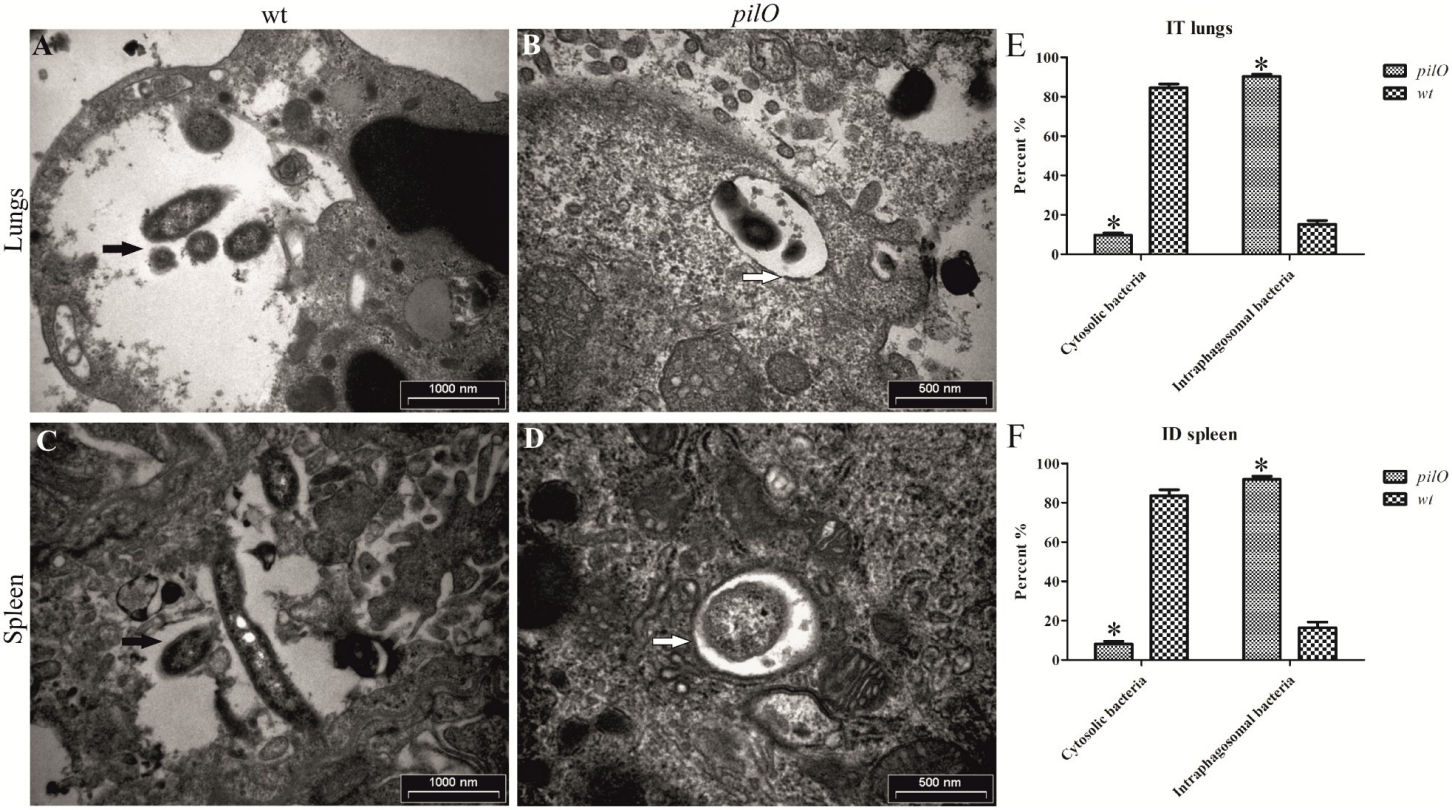
Ultrastructure of lungs and spleen tissue of infected BALB/c mice. Ultrastructure of organs was analysed by transmission electron microscopy (TEM) at 72 h post infection, to estimate the integrity of phagosomal membranes and the intracellular localization of bacteria. Shown are micrographs of the lung tissue of animals infected intratracheally (i.t.) with wt *F*. *novicida* **(A)** or the *pilO* mutant strain **(B)**, as well as the ultrastructure of spleen tissue of BALB/c mice after intradermal (i.d.) infection with wt *F*. *novicida* **(C)** and the *pilO* mutant strain **(D)**. Black arrows show bacteria, while white arrows show intact phagosomal membranes. Quantitative analyses were performed to assess the degree of cytosolic or intraphagosomal bacteria within the lungs of i.t. infected mice **(E)**, or in the spleen of i.d. infected mice **(F)**. Results are obtained by counting at least 100 bacteria for each sample. Asterisks denote that the localization of the *pilO* mutant is statistically different from that of wt *F*. *novicida*, as determined by Student t- test (*, p<0.05).

Ultrastructural analyses of lung tissue after i.t. infection with the wt strain of *F*. *novicida* revealed that bacteria were located intracellulary, predominantly within alveolar macrophages. About 85% of wt bacteria were replicating free in the cytosol of macrophages, with no residual surrounding phagosomal membrane (black arrows) (Fig 10A and 10E). In contrast to the wt strain, 90% of the *pilO* mutant bacteria were single and still enclosed in an intact phagosome within the cells (white arrows) (p<0.05) (Fig 10B and 10E).

Similar results were obtained upon i.d. infection of mice. At 72 h after infection, spleens of *Francisella*-infected mice were harvested and prepared for ultrastructural analysis by TEM. At that time point, 84% of intracellular wt were found in the cytoplasm of the splenocytes with no visible surrounding membranes (black arrows) (Fig 10C and 10F). In contrast, 92% of the *pilO* mutant bacteria were enclosed in an intact phagosome within the splenocytes of BALB/c mice (white arrows) (p<0.05) (Fig 10D and 10F).

We conclude that within both pulmonary cells and splenocytes of BALB/c mice, wt *F*. *novicida* disrupts the phagosomal membrane followed by escape into the cytosol. In contrast, the *pilO* mutant is impaired in phagosomal escape within the lung cells and splenocytes of BALB/c mice. Altogether, these data showed that PilO is essential for phagosomal escape of *F*. *novicida* into the cytosol of the pulmonary cells and splenocytes of BALB/c mice.

## Discussion

*F*. *tularensis* is highly virulent, facultative intracellular bacterium that causes tularemia in humans and animals. The initial step for *Francisella* to reach intracellular environment and finally cause a disease is the process of attachment to the host cells. It is known that *Francisella* uses a variety of adhesins and receptors, however, specific adhesin receptor interactions that contribute to virulence are not yet described. The ability to survive and replicate within the host cells, especially in macrophages, represents a major virulence mechanism of *F*. *tularensis* [59]. Some mutants that are impaired in intracellular survival are also attenuated for virulence in mouse model [25,60]. Remodeling biogenesis of the FCP and subsequent bacterial escape into the cytosol are a key mechanisms of manipulation of the host cell and manifestation of tularemia [61]. Intracellular bacteria, including *Francisella*, use the T6SS which appears to be essential for bacterial phagosomal escape and intracellular replication [32]. *Francisella* uses a non-canonical T6SS, encoded by the FPI, which is a highly dynamic nanomachine assembled on the cell pole, capable of delivering effector proteins across target cell membranes of both bacterial and eukaryotic cells and is often required for bacterial pathogenesis [62–65]. The secretion of effector proteins PdpA, IglC, IglE and PdpE via T6SS was detected by both *F*. *tularensis* and *F*. *novicida* [54].

Type IV pili (T4P) are multifunctional protein fibers produced on the surfaces of a wide variety of bacteria, including *Francisella*. The major subunit of T4P is the type IV pilin and other structurally related proteins, which are included in multiple other functions, such as motility, attachment to chemically diverse surfaces and secretion of a multiple structurally distinct protein substrates [66].

Previous studies showed that type IV pili of *Francisella* are not required for intracellular replication within murine BMDM, A549 or FL83B cells [43], however the *pilE1* mutant is attenuated for replication within J774.1 macrophage cell line [34]. Mutations in the *pilF*, *pilT* and *pilE4* genes of *F*. *novicida* eliminate the pilus expression, while *pilF* gene of this bacterium is also required for protein secretion [34]. Some of the *pil* genes (*pilB*, *pilC*, *pilQ* and *pilA*) of *F*. *novicida* have been shown to be required for protein secretion in rich medium [47]. Until now, the *Francisella pil* mutants have not been tested for replication within human macrophages, and the function of the PilO protein in *Francisella* is still largely unknown.

In *Pseudomonas*, the PilO protein is essential for the process of glycosylation. The *pilO* mutant of *Pseudomonas* still expresses surface pili and exhibit twitching motility, suggesting that glycosylation is not necessary for pili function [66].

Type IV pili proteins have been shown to be involved in a broad range of functions, although their exact biological roles still remain to be determined [66]. It is interesting how Pili proteins with widely divergent sequences can play such similar roles, and how, in some cases, single residue changes in otherwise identical proteins can significantly affect their function [67,68]. In some bacteria, T4P have multiple functions, for example, the T4P of *Neisseria* act as both motility organells and adhesins, while the corresponding pili system in *V*. *cholerae* is involved in adherence and protein secretion [66–68]. Bioinformatic analysis of sequenced genomes have shown a great diversity of genes with the potential to encode pili-associated proteins. In addition, the variety of processes in which these proteins are involved is surprisingly diverse, and require further research [69].

Our results showed that PilO of *F*. *novicida* is required for pilus assembly on the surface of bacterium. Besides that, the *pilO* mutant showed impaired ability to adhere to the surface of HMDMs. Further, using *in vitro* KCl secretion assay we tested if the deletion of the *pilO* in *F*. *novicida* affects the secretion of the T6SS effector protein IglC. The IglC was successfully secreted into culture supernatants of *F*. *novicida* and complemented strain *pilO/O* but not in the supernatant of the *pilO* mutant, indicating that the PilO protein is required for normal IglC export. The inability of the *pilO* mutant to secrete the IglC protein suggest the involvement of the PilO protein in assembly or function of the T6SS machinery. Even though the amount of the proteins loaded on the gel was the same for all bacterial strains, we observed notably lower levels of the IglC and PdpB proteins for the *pilO* mutant, in comparison to the wt *F*. *novicida* and the complemented strain. Since our results did not indicate any growth defect of the *pilO* mutant, it is possible that the loss of the PilO protein leads to decreased expression levels of the IglC and PdpB proteins. Whether the phenotype also includes other FPI proteins and can be ascribed to direct defects on transcription, translation and/or protein stability requires further investigation.

Further, following bacterial intracellular trafficking we determined that the *pilO* mutant is defective for phagosomal escape and intracellular replication within the HMDMs. The obtained results are consistent, since the functional T6SS is known to be required for phagosomal escape and intracellular replication of *Francisella* [32].

*F*. *novicida*, although avirulent for humans, is highly virulent in BALB/c and C57BL/6 mice causing a severe tularemia [4,28,70–73]. The severity of the disease is dependent on mice and bacterial model strain as well as on the route of infection [74–76]. Previous experiments showed that pili are virulence determinants important for pathogenesis in mice by the intradermal route [43]. Among the *pil* mutants of *F*. *novicida*, *pilA* and *pilF* showed reduced virulence in mice [34,43]. *F*. *tularensis* LVS *pilE5*, *pilE6* and *pilT* mutants are attenuated in mice [77]. The *pilE1* mutant of *F*. *tularensis* subsp. *holarctica* is severely attenuated for virulence in mice inoculated by subcutaneous route [78]. Further, a strain of *F*. *novicida* lacking *pilE4* replicates efficiently within macrophages but is slightly attenuated for virulence in mice [34]. Consistent with previous studies showing that mouse mortality after *F*. *tularensis* LVS infection is highly dependent on the bacterial dose as well as the route of infection [58], our results indicate that BALB/c mice are more susceptible to i.t. than i.d. infection with *F*. *novicida*. Further, bacterial burdens of *F*. *novicida* in organs of mice were significantly greater after i.t. than i.d. infection, despite of 1000-fold lower inoculation dose by i.t. route. In contrast to the wt strain, the *pilO* mutant strain showed to be attenuated for virulence in BALB/c mice. Similar to the *pilA* and *pilF* mutants which exhibit reduced virulence in the mouse model [34,43], the *pilO* mutant is severely defective for intracellular replication in the lungs, liver and spleen of BALB/c mice. In contrast to the wt strain, *pilO* mutant doesn’t cause any changes within the organs of mice. In addition, the ultrastructural analyses of lungs and spleen tissue of infected BALB/c mice showed the localization of *pilO* mutant within the intact phagosomal membrane in alveolar macrophages and splenocytes. Single *pilO* mutants stay enclosed in an intact phagosome within the cells.

Within this paper, we show for the first time that the *pilO* gene of *F*. *novicida* is essential for pilus assembly, adherence to the host cell, secretion of putative effector proteins via T6SS and subsequently for intracellular replication of this bacterium in HMDMs, as well as for pathology of tularemia in BALB/c mice.

## Supporting information

S1 Raw Data **Gene expression of *pilM*, *pilN*, *pilO*, *pilP* and *pilQ*** was detected by real time quantitative PCR in mutant, complemented and WT strain using 7500 Fast Real-Time PCR System. Expression level of genes was analyzed relative to wild-type expression using the 2^-ΔΔCT^ method. The error bars represent standard deviations of three independent biological replicates. Asterisks denote that the gene expression of the complemented strain is statistically different from that of the *pilO* mutant, as determined by Student t- test (*, p<0.05).
(TIF)

S2 Raw Data The presence of IglC and PdpB in the concentrated supernatants or bacterial pellets of *F*. *novicida*, the *pilO* mutant, the complemented strain *pilO/O* and *iglC* mutant grown in the presence or absence of 5% KCl. (TIF)

S3 Raw Data Coomassie staining. The concentrated supernatants or bacterial pellets of *F*. *novicida*, the *pilO* mutant, the complemented strain *pilO/O* and *iglC* mutant grown in the presence or absence of 5% KCl were separated using SDS-PAGE and visualized by Coomassie blue staining.
(TIF)
