# Supplementary figures and images for: The type IV pili component PilO is a virulence determinant of Francisella novicida
Source: PLoS One. 2022 Jan 25;17(1):e0261938. doi: 10.1371/journal.pone.0261938 (PMC8789160; doi:10.1371/journal.pone.0261938)

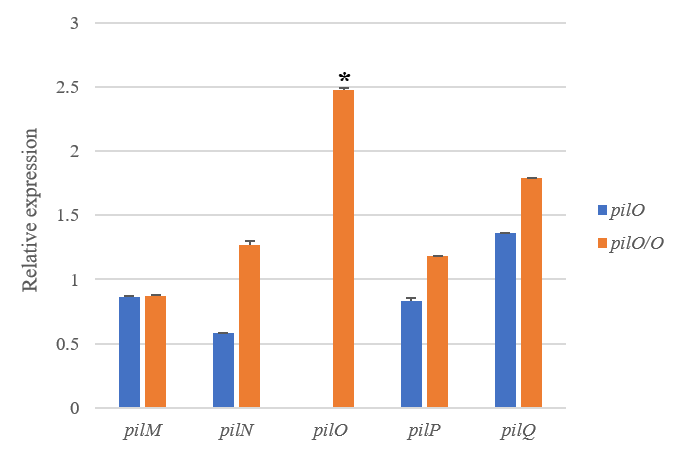

Supplement: S1 Raw Data — Gene expression of pilM, pilN, pilO, pilP and pilQ was detected by real time quantitative PCR in mutant, complemented and WT strain using 7500 Fast Real-Time PCR System. Expression level of genes was analyzed relative to wild-type expression using the 2-ΔΔCT method. The error bars represent standard deviations of three independent biological replicates. Asterisks denote that the gene expression of the complemented strain is statistically different from that of the pilO mutant, as determined by Student t- test (*, p<0.05). (TIF) [file pone.0261938.s001.tif]

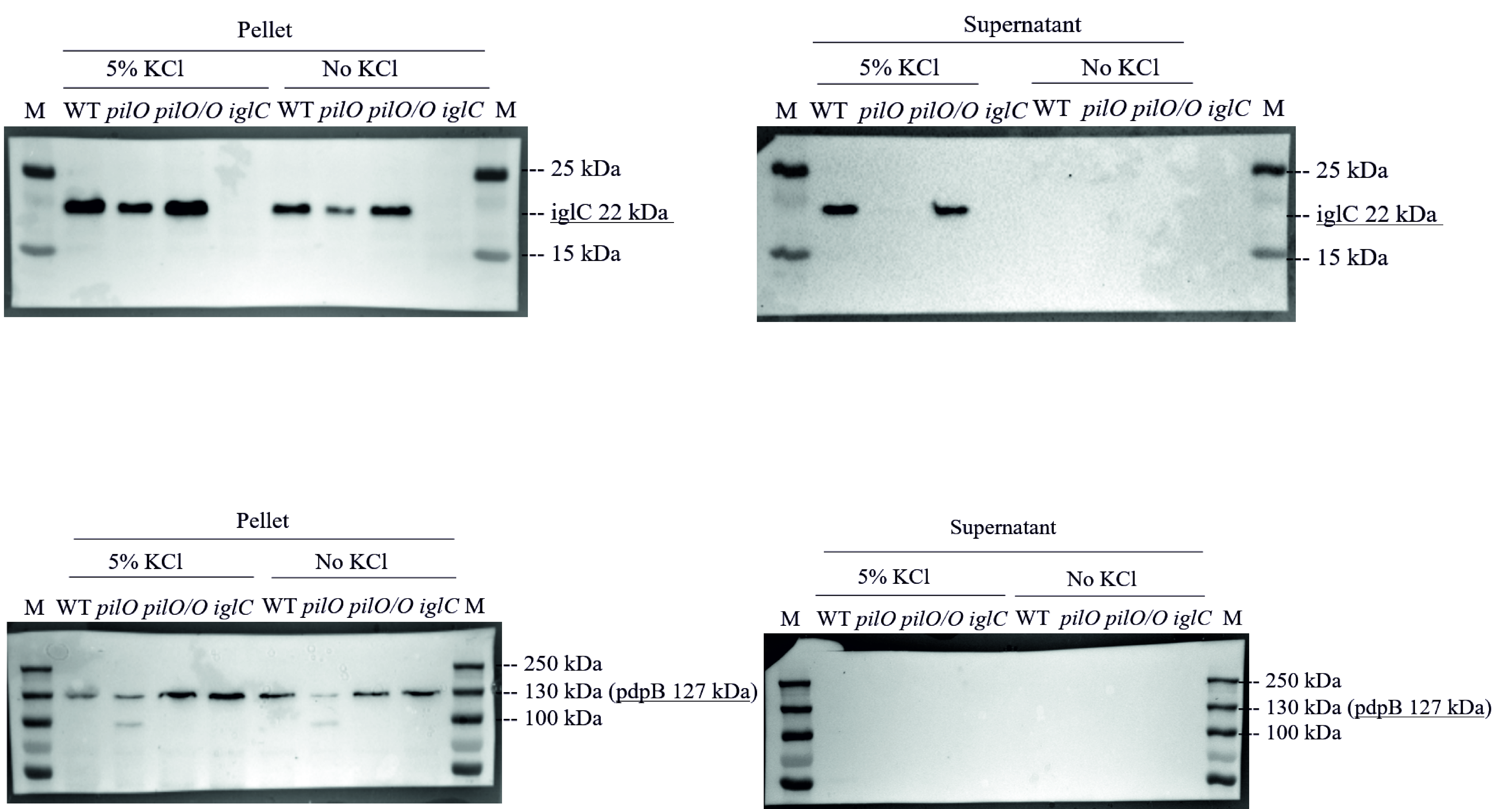

Supplement: S2 Raw Data — (TIF) [file pone.0261938.s002.tif]

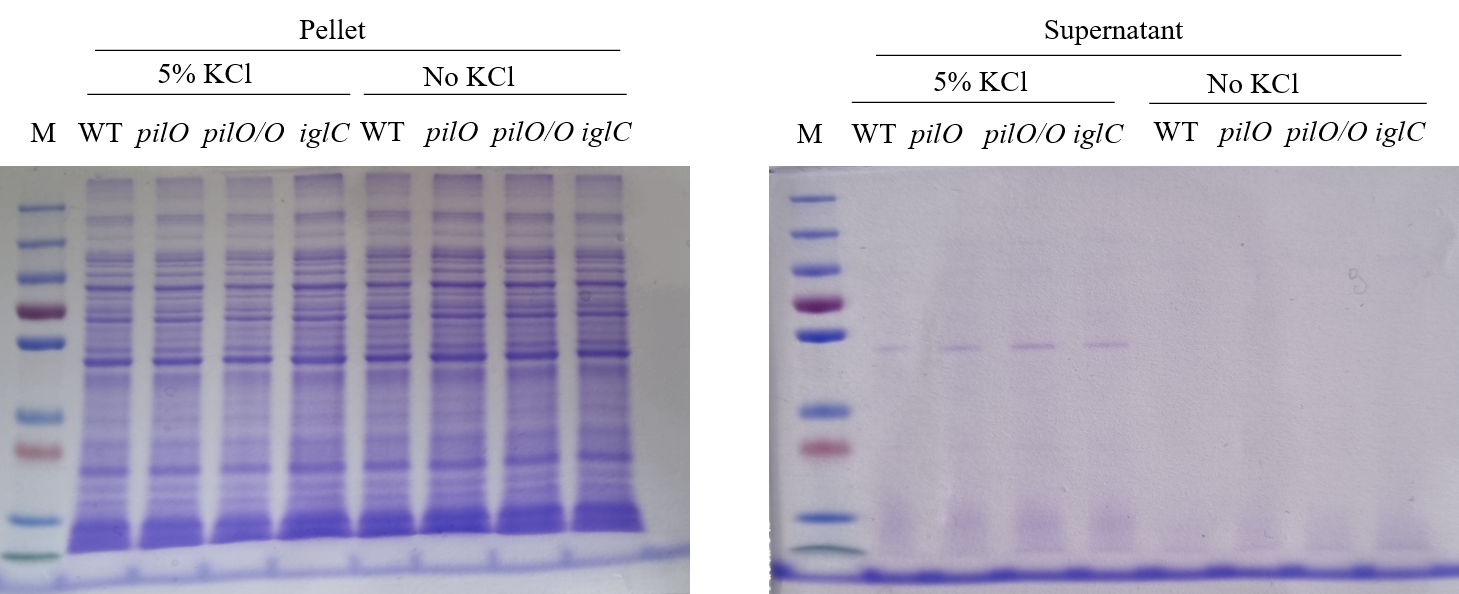

Supplement: S3 Raw Data — The concentrated supernatants or bacterial pellets of F. novicida, the pilO mutant, the complemented strain pilO/O and iglC mutant grown in the presence or absence of 5% KCl were separated using SDS-PAGE and visualized by Coomassie blue staining. (TIF) [file pone.0261938.s003.tif]
